# Calibration of a detector array through beam profile reconstruction with error‐locking

**DOI:** 10.1120/jacmp.v15i6.4591

**Published:** 2014-11-08

**Authors:** Song Wang, Zhiqiu Li, K.S. Clifford Chao, Jenghwa Chang

**Affiliations:** ^1^ Radiation Oncology Weill Cornell Medical College, Cornell University New York NY; ^2^ Radiation Oncology College of Physicians and Surgeons, Columbia University New York NY; ^3^ Radiation Oncology New York‐Presbyterian Hospital New York NY USA

**Keywords:** sensitivity calibration, array calibration, propagation error, MapCHECK, EPID

## Abstract

An iterative method is proposed to calibrate radiation sensitivities of an arbitrary two‐dimensional (2D) array of detectors. The array is irradiated with a wide open‐field beam at the central position, as well as at laterally and longitudinal shifted positions; the 2D beam profile of the wide field is reconstructed iteratively from the ratios of shifted images to the central image. The propagation errors due to output variation and inaccurate array positioning are estimated and removed from the reconstructed beam profile by an error‐locking scheme with narrow open‐field irradiations. The beam profile is interpolated when necessary and then compared to raw detector responses to determine sensitivities. Two additional methods were implemented for comparison: 1) the commercial iterative calibration method for MapCHECK2 with translation and rotation operations; 2) a labor‐intensive noniterative method without the issue of error propagation. A MapCHECK2 2D detector array was used to validate the proposed method with the 6 MV photon beam from a Varian iX linear accelerator. All calibration methods were repeated three times. A total of 5, 9, and 29 irradiations were required to implement the commercial method, the proposed method and the noniterative method respectively. Moreover, a 5 mm positioning error was intentionally introduced into the calibration procedures of the commercial and the proposed method to test their robustness. Under the normal operation condition of the linear accelerator and with careful alignment of the MapCHECK2, the deviations of the calibrated sensitivities of the proposed method and commercial method with respect to the noniterative method were 0.30%±0.29% and 0.92%±0.63% respectively; when the 5 mm positioning error was presented, these two methods resulted in deviations of 0.40%±0.36% and 3.58%±1.94%, respectively. A patient study suggested that, due to this 5 mm positioning error, the mean DTA (dose to agreement) passing rate by the commercial method was 2.7% lower than that by the noniterative method, whereas the proposed method led to a comparable passing rate. It is evident from this study that the proposed iterative method leads to within 1% mean calibration results to established methods. It requires much fewer number of measurements than noniterative method and is more robust against the positioning error than the commercial iterative method. The method also eliminates the need of rotation operations and, therefore, is applicable to inline detector arrays without rotation function, such as electronic portal imager device (EPID).

PACS number: 87.56.Fc

## I. INTRODUCTION

Special calibration procedure is required when a 2D detector array is used for dosimetry purpose. Since the radiation sensitivities (detector responses to unit radiation fluence) of the detectors in a 2D array are not identical, each detector requires an individual calibration factor. However, it is impractical to irradiate one detector at a time with unit fluence. Therefore, a wide open‐field beam could be used to irradiate the whole 2D array so that the sensitivity of each detector can be derived from the raw detector readings and the fluence of the irradiated field; the fluence is usually not uniform and needs to be modified or determined, as well.

The sensitivities of detectors are simply the raw detector readings from the irradiation of an ideally flat beam. Parent et al.(1) proposed to shift and irradiate a small squared open‐field beam multiple times to cover the active region of the detector array (multiple field approach, or MF). By assuming uniform fluence within the small field, this is equivalent to delivering a virtual wide open‐field beam with uniform fluence. This method requires many small fields to irradiate the whole detector array. For instance, $16\;10\times 10\;{\text{cm}}^{2}$ fields were irradiated to calibrate an iViewGT electronic portal imaging device (EPID). The fluence of a wide open field can also be artificially made uniform by placing a solid water slab in the beam path, as suggested by Siebers et al.^(2)^ (solid water approach, or SW). The thickness of the slab is determined by Monte Carlo (MC) simulations involving modeling the open‐field beam generation from a linear accelerator (linac), beam transmission through the solid water slab, and energy‐dependent detector responses of the 2D array.

Given a known delivered beam profile, the sensitivities can be calculated by comparing this beam profile to the raw detector response. This category of approaches is contingent upon accurately estimating/reconstructing sensitivity‐free beam profile. For instance, a third‐order polynomial can be used to fit the one‐dimensional (1D) MC‐simulated wide open‐field beam profile (1D MC fitting approach, or 1DMCF), and the delivered open‐field beam profile can, therefore, be represented by this polynomial along with a function describing the jaw settings of the field. Field asymmetry can be further modeled by the 2D diode array measurements. The proposed method, again by Parent et al.(3) resulted in less than 3% sensitivity deviations from MF and SW methods. In a separate approach by Greer,(4) a 1D wide open‐field beam profile is extracted from the wide beam measurement with sensitivities estimated by irradiating a small rectangular open‐field beam repeatedly to different lateral locations of the detector array (1DBP approach). The 2D beam profile is completely represented by this 1D beam profile with the assumption that the 2D beam profile is perfectly symmetrical. For example, to calibrate a Varian aS500 EPID, a $40\times 30\;{\text{cm}}^{2}$ wide open‐field beam was irradiated once and a $10\times 30\;{\text{cm}}^{2}$ small open‐field beam was irradiated 11 times with a lateral shifting step of 2.5 cm. The reconstructed beam profile has to be interpolated from a resolution of 2.5 cm to that of detector size (∼0.8mm for aS500) in order to calculate detector‐by‐detector sensitivities.

The method by Simon et al.^(5)^ requires neither fluence modification nor beam profile reconstruction. In his approach, the detector array needs to be translated and rotated 90° and 180° with repeated irradiation of a wide beam, and sensitivities are obtained iteratively from the ratio image of the central and translated measurements. The propagation errors are estimated from the central and the 180° rotated measurements. The above method and its variants are patented and used to calibrate Profiler, MapCHECK, and other 2D array products. It will be referred to as “MapCheck_TR” hereinafter. This method is, by far, the most sophisticated iterative method in the array calibration with propagation error analysis and reduction. Donetti et al.^(6)^ proposed a similar approach, but it is limited to a square array calibration.

In another major category of approaches, the detector array is irradiated by a wide open‐field beam and the response is compared to a reference measurement to determine the sensitivities. The reference measurement is acquired by another calibrated single dosimeter or detector array. Taking Varian's portal dosimetry^(7)^ as an example, EPID is irradiated by a wide field beam and raw detector readings are compared to in‐water beam profile to derive pixel‐by‐pixel calibration factors. It is noteworthy that, strictly speaking, these factors are not sensitivities, as the radiation response of EPID is not water equivalent. This is also generally true for similar approaches in which the investigated 2D array has a different radiation characteristic from the reference dosimeter.

In this study, we developed a two‐dimensional beam profile reconstruction method (2DBP) to improve the efficiency and accuracy of 2D array sensitivity calibration. The proposed method reconstructs the sensitivity‐free 2D beam profile of a wide open‐field beam first and then uses the reconstructed beam profile for the array calibration. Beam asymmetry and tilting are handled implicitly by this method; these are only considered in 1DMCF and MapCHECK_TR methods. The proposed method does not require complex MC modeling, as in SW and 1DMCF methods, and is completely based on measurements. Rotation of the detector array, such as in the MapCHECK_TR method, is completely avoided in this method so that it can be potentially applied to a detector array without rotation function, such as EPID. Potential calibration errors are analyzed and modeled in this study; a novel error‐locking approach is developed to remove the errors, as well. It will be demonstrated later in this study that the proposed approach can remove the calibration error from inaccurate positioning that can't be handled by the MapCHECK_TR method. We will compare and validate the proposed method with the MapCHECK_TR method (the standard commercial calibration method for MapCHECK2) and the 2D beam profile method (2DBP_Greer) extended from Greer's 1DBP method.

## II. MATERIALS AND METHODS

### A. Materials

Experiments of this study were conducted on MapCHECK2 (Sun Nuclear Corporation (SNC), Melbourne, FL). Diode detectors in MapCHECK2 are distributed on a $26\times 32\;{\text{cm}}^{2}$ (row by column) area, but there is no detector in four $7\times 7\;{\text{cm}}^{2}$ corner triangles. Detectors on each row have a 1 cm lateral spacing and the row‐to‐row interval is 0.5 cm. There is also a 0.5 cm lateral shifting between neighboring rows, leading to a diagonal detector‐to‐detector distance of about 0.7 cm. In this study, the “un‐shifted” MapCHECK2 is referred to as a MapCHECK2 with the center of the device aligned to the central axis (CAX) of the linac; the “shifted” MapCHECK2, referred to as the center of the device, is shifted away from the CAX of the linac. The shifting is limited to the lateral and longitudinal directions. The distance between the radiation source and the detector plane is always 100 cm. The 2D beam profile is reconstructed on about half of the total detectors that are on a rectangular 2D grid of 1 cm spacing with the center of the grid overlying the center of the detector array; the beam profiles on other rows of detectors (with a 0.5 cm lateral and longitudinal offsets from aforementioned detectors) can be interpolated from the reconstructed beam profile.

All calibration fields were delivered by a Varian iX linac with a beam accelerating potential of 6 MeV for 200 MU at a dose rate of 300 MU/min. Gantry and collimator angles were fixed to 0° throughout the experiments. During the calibration, there was no buildup material placed on the MapCHECK2. The software used to record and save the measurements was SNC patient version 6.2.2. Each measurement was saved into a text file in a vendor‐defined format with sections corresponding to 2D raw detector responses, calibration factors, and other information. The 2DBPGreer and the proposed method were implemented in MATLAB version R2009b (MathWorks, Natick, MA). The saved measurement files were parsed and fed to the MATLAB routines as inputs. The recalculated sensitivities and recalibrated raw data can be written to a text file in the vendor's format in order to analyze it in the SNC patient software.

### B. Two‐dimensional beam profile reconstruction and sensitivity calculation

The 2D in‐detector beam profile, R(X,Y), is reconstructed in the following manner from data measured with a 2D detector array. The array measurement when aligned to the CAX of the beam is:

(1) $$M_{C}(X,Y)=R(X,Y)\times S(X,Y)$$

where S(X,Y) is a matrix of the detector sensitivities; *X* refers to lateral distance from the center of a 2D detector or from the CAX of a 2D beam profile; *Y* refers to the longitudinal distance; and one unit of X or Y corresponds to the shifting distance of the 2D detector array in the measurement steps described in next two subsections and must be the integer multiplication of the detector spacing. (X,Y) is located in the detector plane with the radiation source to plane distance of 100 cm, to be consistent with the measurement setup in this study.

#### B.1 Measurement along the x‐axis (lateral)

**Figure 1:**
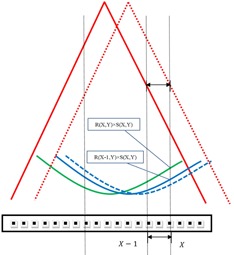
Reconstruction of the 1D lateral beam profile RX(X,Y) on the row Y of the detector array from unshifted measurement $M_{C}(X,Y)$ and shifted $M_{\text{LAT}–1}(X,Y)$. Solid red lines represent the diverging wide‐beam edges of unshifted wide‐field irradiation and dashed red is the shifted. Solid green and solid blue curves are unshifted and shifted beam profiles. These curves represent the typical bowl‐shaped beam profiles from the Varian iX linac photon beams; penumbras are not drawn as the wide field used for calibration usually is larger than the detector array to avoid irradiation from high‐gradient penumbras. Black rectangle is the representation of a cross section of a 2D array and black dots are individual detectors. As illustrated, an arbitrary detector X reads out radiation signal R(X,Y)×S(X,Y) and R(X–1,Y)×S(X,Y) from unshifted and shifted irradiation, respectively. The sensitivity term in the ratio of two measurements, R(X,Y)/R(X–1,Y), is cancelled out and therefore the ratio can be used to reconstruct the sensitivity‐free beam profile. However, if there is a positioning error and suppose the recorded shifted measurement is the dashed blue beam profile as in the figure, the recorded values would be higher and lower than the expected values on the left‐hand and right‐hand side, respectively. The ratios of two measurements will change accordingly and result in flatter or sharper reconstructed beam profile than the expected one in terms of the Eqs. (15), (16).

The detector array is shifted laterally to the left with respect to the CAX of the beam by the distance of one column or several columns of detectors, as illustrated in Fig. 1. The array measurement is as follows:

(2) $$M_{\text{LAT}-1}(X,Y)=R(X-1,Y)\times S(X,Y)$$

since the array detectors at positions (X,Y) are now under beam profile position (X‐1,Y).

From Eqs. (1), (2) one has:

(3) $$R(X-1,Y)=R(X,Y)\times \left(\frac{M_{\text{LAT}-1}(X,Y)}{M_{C}(X,Y)}\right)$$

From Eq. (3) for position X in the beam profile one has:

(4) $$R(X,Y)=R(X+1,Y)\times \left(\frac{M_{\text{LAT}-1}(X+1,Y)}{M_{C}(X+1,Y)}\right)$$

From Eqs. (3), (4) one has:

(5) $$R(X-1,Y)=R(X+1,Y)\times \prod_{i=0}^{i=1}\left(\frac{M_{\text{LAT}-1}(X+i,Y)}{M_{C}(X+i,Y)}\right)=R(X+1,Y)\times \prod_{i=1}^{i=2}\left(\frac{M_{\text{LAT}-1}(X-1+i,Y)}{M_{C}(X-1+i,Y)}\right)$$

The general iteration equation in the lateral direction can now be written as follows:

(6) $$R(X1,Y)=R(X2,Y)\times \prod_{i=1}^{i=X2-X1}\left(\frac{M_{\text{LAT}-1}(X1+i,Y)}{M_{C}(X1+i,Y)}\right)\;\text{for}\;X2>X1$$

Normalization of any beam profile position (X,Y) is done as follows:

(7) $$RX_{\text{NOR}}(X,Y)=\left(\frac{R(X,Y)}{R(0,Y)}\right)$$

Each row of ${\text{RX}}_{\text{NOR}}(X,Y)$ represents a relative 1D lateral beam profile normalized to its center.

If X<0, then from Eq. (6) one has:

(8) $$\begin{array}{l} R(X,Y)=R(0,Y)\times \prod_{i=1}^{i=-X}\left(\frac{M_{\text{LAT}-1}(X+i,Y)}{M_{C}(X+i,Y)}\right)\\ RX_{\text{NOR}}(X,Y)=\prod_{i=1}^{i=-X}\left(\frac{M_{\text{LAT}-1}(X+i,Y)}{M_{C}(X+i,Y)}\right) \end{array}$$

If X>0, then from Eq. (6) one has:

(9) $$\begin{array}{l} R(0,Y)=R(X,Y)\times \prod_{i=1}^{i=X}\left(\frac{M_{\text{LAT}-1}(i,Y)}{M_{C}(i,Y)}\right)\\ RX_{\text{NOR}}(X,Y)=\frac{1}{\prod_{i=1}^{i=X}\left(\frac{M_{\text{LAT}-1}(i,Y)}{M_{C}(i,Y)}\right)}=\prod_{i=1}^{i=X}\left(\frac{M_{C}(i,Y)}{M_{\text{LAT}-1}(i,Y)}\right) \end{array}$$

#### B.2 Measurement along the y‐axis (longitudinal)

The array is shifted longitudinally to the inferior with respect to the CAX of the beam by the distance of one row or several rows of detectors, and then the array measurement is as follows:

(10) $$M_{\text{LNG}-1}(X,Y)=R(X,Y-1)\times S(X,Y)$$

Since the array detector at position (X,Y), is now under beam profile position (X,Y‐1).

Similar to the derivation of the relative 1D lateral beam profile, the longitudinal profile can be reconstructed from $M_{\text{LNG}–1}(X,Y)$ and $M_{C}(X,Y)$ as follows:

If Y<0, one has:

(11) $$RY_{\text{NOR}}(X,Y)=\prod_{i=1}^{i=-Y}\left(\frac{M_{\text{LNG}-1}(X,Y+i)}{M_{C}(X,Y+i)}\right)$$

If Y>0, one has:

(12) $$RY_{\text{NOR}}(X,Y)=\frac{1}{\prod_{i=1}^{i=Y}\left(\frac{M_{\text{LNG}-1}(X,i)}{M_{C}(X,i)}\right)}\prod_{i=1}^{i=Y}\left(\frac{M_{C}(X,i)}{M_{\text{LNG}-1}(X,i)}\right)$$

#### B.3 Sensitivity calculation

The relative 2D beam profile can be obtained by reweighing relative 1D lateral beam profiles with central 1D longitudinal profile as

(13) $$R_{\text{NOR}}(X,Y)=RX_{\text{NOR}}(X,Y)\times RY_{\text{NOR}}(0,Y)$$

The reconstructed 2D beam profile might need to be interpolated to $r_{\text{nor}}(x,y)$, where (x,y) corresponds to a physical detector position. When an array is shifted by a distance more than the physical detector spacing, the 2D beam profile is reconstructed on a grid with a resolution coarser than that of physical detectors. An interpolation needs to be performed to calculate the beam profile at finer resolution in order to calculate detector‐to‐detector sensitivities. Moreover, the beam profile is always reconstructed on a regular (rectangular or square) grid by the proposed method. An interpolation is also necessary to calculate the beam profile at those detector positions that can't be represented by the regular grid. The latter is the case of MapCHECK2 calibration.

Then, the calculation of the sensitivities is straightforward:

(14) $$s(x,y)=\frac{m_{c-\text{nor}}(x,y)}{r_{\text{nor}}(x,y)}$$

where s(x,y) is the detector‐to‐detector sensitivity matrix and $m_{c–\text{nor}}(x,y)$ is the unshifted raw array measurement, normalized to the central detector response.

### C. Error analysis

In above derivation of beam profile reconstruction, we hypothesize that the delivered beam profile remains unchanged during the repeated measurements and the detector array can be perfectly positioned. However, this does not reflect the measurement condition. The Eqs. (8), (9) are rewritten with error‐modeling terms as follows, respectively:

(15) $$\begin{array}{l} RX_{\text{NOR}}^{'}(X,Y)=\prod_{i=1}^{i=-X}\left(\frac{M_{\text{LAT}-1}(X+i,Y)}{M_{C}(X+i,Y)}\right)\times {\left(1+E1-E2\right)}^{-X}\\ =RX_{\text{NOR}}(X,Y)\times {\left(1+E1-E2\right)}^{-X}\;\text{for}\;X<0 \end{array}$$

and

(16) $$\begin{array}{l} RX_{\text{NOR}}^{'}(X,Y)=\prod_{i=1}^{i=X}\left(\frac{M_{C}(i,Y)}{M_{\text{LAT}-1}(i,Y)}\right)/{\left(1+E1+E2\right)}^{X}\\ =RX_{\text{NOR}}(X,Y)/{\left(1+E1-E2\right)}^{X}\;\text{for}\;X>0 \end{array}$$

where ${\text{RX}}_{\text{NOR}}(X,Y)$ is the ideal normalized beam profile and ${\text{RX}}_{\text{NOR}}^{\prime }(X,Y)$ is the one with propagation error; E1 is the output variation of the shifted measurement $M_{\text{LAT}–1}$ with respect to the unshifted measurement $M_{C}$. This error alone will have different effect on two sides of the reconstructed beam profiles, as described by Eqs. (15), (16). For instance, if *E*1 is positive, ${\text{RX}}_{\text{NOR}}^{\prime }(X,Y)(X>0)$ will be decreased while ${\text{RX}}_{\text{NOR}}^{\prime }(X,Y)(X<0)$ will be increased, leading to a tilted, asymmetrical reconstructed beam profile; *E*2 is due to the positioning error and is in close connection to the shape of the beam profile as the error is dependent of the gradient. For instance, the linac beam profile has a bowl‐like shape and is ascending towards the beam edges. When there is a positioning error along the shifting direction during the calibration, it would be higher than the expected on one side and lower on the other side, as illustrated in Fig. 1. This error is, therefore, modeled with different signs. In terms of Eqs. (15), (16), this error alone leads to the reconstructed beam profile flatter or sharper than expected and the behavior is almost symmetrical. For instance, if *E*2 is positive, ${\text{RX}}_{\text{NOR}}^{\prime }(X,Y)(X>0)$ will be decreased and ${\text{RX}}_{\text{NOR}}^{\prime }(X,Y)(X<0)$ will be decrease, as well. The positioning error is modeled as a single factor whereas it actually varies with respect to the local gradient. This simplified model estimates the positioning error on an average sense.

It is noteworthy that the error terms in Eqs. (15), (16) depend on the reconstruction position X; the greater distance the reconstruction position is from the center of the beam profile, the bigger the error. In other words, the error accumulates toward the beam edges. The errors at an individual reconstruction position might be small, but the accumulated error might not be negligible.

In the MapCHECKTR method, the local error is estimated using Eq. (25) of Simon's patented method.^(5)^ This equation is duplicated as follows:

(17) $$\left(\prod_{i=1}^{E-1}\frac{C_{i}}{D_{i+1}}\right)\times (D_{\text{dc}}\times {S_{\text{dc}})}^{E-1}=\sqrt{\frac{C_{1}}{C_{E}}\times \frac{A_{1}}{A_{E}},}$$

where *C* and *D* are unshifted and shifted measurements, and $D_{\text{dc}}\times S_{\text{dc}}$ is position‐invariant error that includes intermeasurement dose and sensitivity variation; definitions of other terms can be found from the cited article. The error model was applied to a linear 1D array indexed from 1 to *E*. The error term $D_{\text{dc}}$ is defined as the ratio of the fluence variation between two measurements. As it is uniformly applied to any detector location, it basically models the output variation. However, as demonstrated in the proposed method, the positioning error contributes to two sides of the beam profile differently and multiplying this error from the detector 1 to detector *E* will basically cancel out the effect of this error. That is, the positioning error can't be detected by the MapCHECKTR method. Therefore, when the proposed method and the MapCHECKTR method are implemented, a special batch of measurements will be conducted with an intentionally introduced 5 mm positioning error to test the robustness of these two iterative methods.

### D. Error‐locking

A procedure is developed to lock the propagation error with additional measurements and is described below for the lateral beam profile reconstruction:

1. A narrow beam is irradiated at three different positions of the detector array. The cross‐sectional measurement geometry is illustrated in Fig. 2 for lateral error‐locking.
  **Figure 2:**
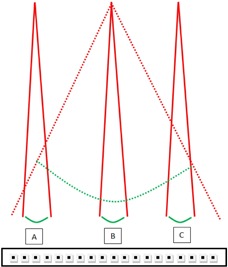
Error‐locking scheme to estimate output and positioning errors. Red lines are the diverging beam edges of irradiations and green curves are corresponding beam profiles. Three narrow open‐field beams are irradiated at the center B and two sides A and C of the 2D array, and one wide open‐field beam is irradiated to the whole detector array. Sensitivities of A and C with respect to B can be calculated since identical fluence is delivered to these locations. Sensitivities can then be used to extract corresponding wide beam profile at A and C and compared to the iteratively reconstructed wide beam profile to estimate errors.
2. On an arbitrary row of detectors, those at points “A”, “B”, and “C” receive identical fluence from the narrow beam. The relative sensitivities of “A” and “C” with respect to “B”, $\frac{S(A,Y)}{S(B,Y)}$ and $\frac{S(C,Y)}{S(B,Y)}$, are known.
3. With acquired relative sensitivities, the sensitivity‐free responses of “A” with respect to “B” in the wide beam measurement $M_{C}(X,Y)$ can be calculated as follows:
  (18) $$\frac{M_{C}(A,Y)}{M_{C}(B,Y)}=\frac{R(A,Y)\times S(A,Y)}{R(B,Y)\times S(B,Y)}$$
  If “B” is the center of the detector array and A<0, the above equation leads to:
  (19) $$RX_{\text{NOR}}(A,Y)=\frac{M_{C}(A,Y)}{M_{C}(0,Y)}/\frac{S(A,Y)}{S(0,Y)}\;A<0$$
  where ${\text{RX}}_{\text{NOR}}(A,Y)$ is the normalized beam profile, as defined in the Material & Methods section B above. Similarly,
  (20) $$RX_{\text{NOR}}(C,Y)=\frac{M_{C}(C,Y)}{M_{C}(0,Y)}/\frac{S(C,Y)}{S(0,Y)}\;C>0$$
4. These responses can be compared to the corresponding responses on the iteratively reconstructed 1D lateral beam profile to calculate *E*1 and *E*2. If X in Eq. (15) is substituted by *A* and ${\text{RX}}_{\text{NOR}}(A,Y)$ is calculated by Eq. (19), the propagation error can be derived as:
  (21) $$(1+E1-E{2)}^{-A}=\frac{R{X^{\prime }}_{\text{NOR}}(A,Y)}{RX_{\text{NOR}}(A,Y)}$$
  Similarly,
  (22) $$(1+E1-E{2)}^{C}=\frac{RX_{\text{NOR}}(A,Y)}{R{X^{\prime }}_{\text{NOR}}(A,Y)}$$
  The errors *E*1 and *E*2 can be obtained by solving Eqs. (21), (22) and can then be used in Eqs. (15), (16) to calculate ${\text{RX}}_{\text{NOR}}(X,Y)$ from ${\text{RX}}_{\text{NOR}}^{\prime }(X,Y)$ at an arbitrary lateral position X.

A similar procedure can be applied to longitudinal error‐locking.

To gauge the calibration errors, the proposed method will be implemented with and without error‐locking; they will be referred to as 2DBP and 2DBPunlocked, respectively, when they need to be differentiated.

### E. Extension of Greer's 1D beam profile reconstruction method (2DBPGreer)

In Greer's 1DBP method, the central‐lateral 1D beam profile ${\text{RX}}_{\text{NOR}}(X,0)$ of a wide beam is reconstructed, with sensitivities extracted by a narrow field scanned laterally. In fact, an arbitrary lateral 1D beam profile ${\text{RX}}_{\text{NOR}}(X,Y)$ having an offset from the central lateral axis can also be reconstructed using the same rationale and existing measurements. Similarly the central‐longitudinal 1D beam profile ${\text{RY}}_{\text{NOR}}(0,Y)$ can be reconstructed and it can be used to reweigh ${\text{RX}}_{\text{NOR}}(X,0)$ to get the 2D beam profile $R_{\text{NOR}}(X,Y)$. Two‐dimensional sensitivities can be derived accordingly. To differentiate this approach from the proposed one, it will be referred to as the 2DBPGreer method. It was implemented in this study as a reference to validate other methods, as it is based on a noniterative approach and reconstruction errors will not propagate.

### F. Patient study

A patient study was conducted to test the robustness of the iterative calibration methods 2DBP and MapCheckTR against an intentionally introduced positioning error during calibration.

The patient plan was made to treat the right thigh and it comprises of seven co‐planar IMRT (intensity‐modulated radiation therapy) fields. The longitudinal lengths of those fields are from 28.5 to 30.5 cm. Those long fields were intentionally selected since the propagation error increases with respect to the distance from the center of the detector array. The longer a field, the more sensitive the field is to the propagation error. Those fields were measured on MapCHECK2 with the sensitivities calibrated by the 2DBP, MapCHECKTR, and 2DBPGreer methods, respectively. The in‐water planar doses were generated by the Pinnacle treatment planning systems (Philips Healthcare, Andover, MA). A DTA (dose to agreement) analysis with 3 mm criteria was conducted between the measured fields and the planar doses in the SNC patient software.

## III. RESULTS

The 2DBPGreer method was implemented on the MapCHECK2 with a wide open‐field beam of $37\times 37\;{\text{cm}}^{2}$ irradiated once, a narrow open‐field beam of $4\times 37\;{\text{cm}}^{2}$ scanned laterally (by shifting the MapCHECK2) from −12cm to 12 cm at a step size of 2 cm, and a narrow open‐field beam of $37\times 4\;{\text{cm}}^{2}$ scanned longitudinally from −16cm to 16 cm at the same step size. The 2D beam profile was reconstructed on a square grid of 2 cm spacing and then interpolated to a resolution at the detector scale. The method was not implemented with 1 cm step size to save time and labor; it otherwise would require 59 instead of 29 irradiations for each calibration. The calibration procedure was repeated three times and the detector‐to‐detector calibration variations among three procedures were calculated. The variation turned out to be 0.19%±0.11%, suggesting excellent agreement of these calibrations.

**Figure 3:**
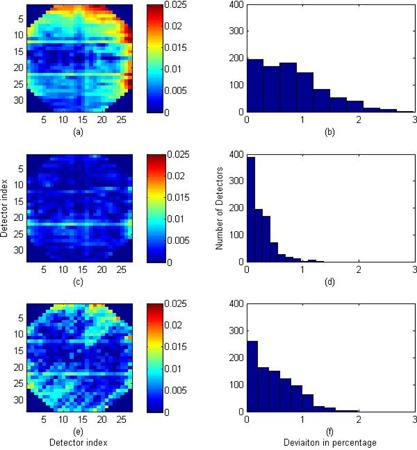
Comparisons of the sensitivity‐corrected 2D beam profiles between the 2DBPGreer method and the 2DBPunlocked, 2DBP, and MapCHECK TR methods, respectively. These results are from the first batch of measurements: (a) the 2D deviations between the 2DBPunlocked and the 2DBPGreer beam profiles; (b) the histogram of up to 3% deviations; (c) the 2D deviations between the 2DBP and the 2DBPGreer beam profiles; (d) the histogram of up to 1.3% deviations; (e) the 2D deviations between the MapCHECKTR and the 2DBPGreer beam profiles; (f) the histogram of up to 1.9% deviations.

The unlocked version of 2DBP (2DBPunlocked) was implemented with a wide open‐field beam of $37\times 37\;{\text{cm}}^{2}$ irradiated repeatedly, with MapCHECK2 unshifted (to measure $M_{C}$), and shifted 1 cm laterally $\left(M_{\text{LAT}–1}\right)$ and longitudinally $\left(M_{\text{LNG}–1}\right)$. The 2D beam profile was reconstructed on a 1 cm grid and then interpolated. The calibration procedure was repeated three times and the absolute difference between the reconstructed beam profile of this method and that of 2DBPGreer for each batch of measurements is tabulated in Table 1. The overall absolute difference from all three batches of measurements is 0.92%±0.63%. The 2D distribution of the differences of the first batch of measurement is shown in the subplot (a) of Fig. 3 and the histogram of the differences is plotted in the subplot (b) of Fig. 3. It can be seen the largest differences are at peripherals of MapCHECK2. This also results in a long tail of the difference histogram.

**Table 1:** The percentage differences of 2D beam profiles between the 2DBP, 2DBPunlocked, and MapCHECKTR methods and the 2DBPGreer method. As the measurements were repeated three times under the normal condition, the mean and standard deviations of differences are calculated for each measurement batch and for accumulated data from all measurement batches, as well. Similar statistical analysis is conducted for the measurement batch with 5 mm positioning error.

*Measurement Batch*	*2DBP*	*2DBPunlocked*	*MapCHECKTR*
#1	0.22±0.22	0.78±0.58	0.48±0.39
#2	0.30±0.31	0.78±0.56	0.90±0.56
#3	0.32±0.37	1.20±0.65	1.14±0.62
Accumulated	0.30±0.29	0.92±0.63	0.84±0.60
5 mm	0.40±0.36	3.33±1.82	3.58±1.94

The locked version of 2DBP (2DBP) was implemented with a lateral error‐locking open‐field beam of $4\times 37\;{\text{cm}}^{2}$ irradiated at −11,0, and 11 cm, respectively, and with a longitudinal error‐locking open‐field beam of $37\times 4\;{\text{cm}}^{2}$ irradiated at −14,0, and 14 cm, respectively. The normalized lateral beam profiles at −11 and 11 cm were calculated using Eqs. (19), (20), and the output and positioning errors were calculated using Eqs. (21), (22). These errors were removed from the iteratively reconstructed beam profiles in terms of Eqs. (15), (16). The longitudinal error‐locking was performed in a similar fashion. The overall absolute difference between the reconstructed beam profile of this method and that of 2DBPGreer is 0.30%±0.29%. The 2D distribution of the differences of the first batch of measurement is shown in the subplot (c) of Fig. 3 and the histogram of the differences is plotted in the subplot (d) of Fig. 3. With error‐locking, the mean and the standard deviation of the differences are significantly reduced.

The MapCHECKTR was implemented according to the manufacturer's instructions and was used to calibrate the MapCHECK2. The overall absolute difference between the reconstructed beam profile of this method and that of 2DBPGreer is 0.84%±0.60%. The 2D distribution of the differences of the first batch of measurement is shown in the subplot (e) of Fig. 3 and the histogram of the differences is plotted in the subplot (f) of Fig. 3.

Each step of the above calibration methods involves realigning the MapCHECK device, delivering irradiation, and saving the result in the SNC patient software. In our experiments, it took on average 2 min to complete one calibration step by two operators. Therefore, the calibration procedures of the 2DGreer, unlocked 2DBP, locked 2DBP, and MapCHECKTR methods took approximately 58, 6, 18, and 10 min, respectively.

**Figure 4:**
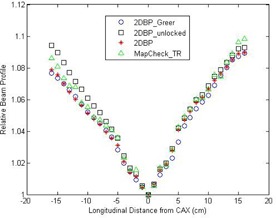
Comparisons of the sensitivity‐corrected 1D longitudinal beam profiles between the 2DBP Greer method and the 2DBP unlocked, 2DBP, and MapCHECKTR methods, respectively. These results are from the first batch of measurements, and the left‐hand side is the gantry side and the right‐hand side is the couch side. CAX = central axis.

The 1D CAX longitudinal beam profiles of all calibration methods from the first batch of calibration measurements are plotted in Fig. 4. It can be seen these profiles are in reasonable agreement, even for the one by the unlocked 2DBP method, suggesting that the output and positioning errors are small. In fact, 1.45% and 0.3% propagation errors are presented on the gantry‐side and the couch‐side of the beam profile by the unlocked 2DBP method, and those corresponding to 0.036% local output error and 0.054% local positioning error in terms of Eqs. (15), (16).

**Figure 5:**
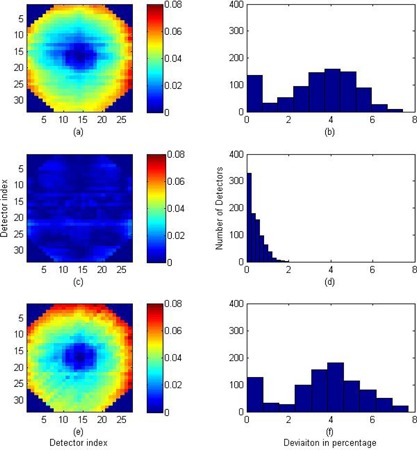
Comparisons of the sensitivity‐corrected 2D beam profiles between the 2DBPGreer method and the 2DBP unlocked, 2DBP, and MapCHECKTR methods, respectively; a 5 mm positioning error is intentionally introduced in the calibration procedures of the latter three methods: (a) the 2D deviations between the 2DBPunlocked and the 2DBPGreer beam profiles; (b) the histogram of up to 7.0% deviations; (c) the 2D deviations between the 2DBP and the 2DBPGreer beam profiles; (d) the histogram of up to 2.0% deviations; (e) the 2D deviations between the MapCHECKTR and the 2DBPGreer beam profiles; (f) the histogram of up to 7.0% deviations.

**Figure 6:**
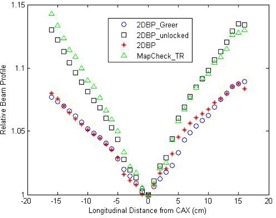
Comparisons of the sensitivity‐corrected 1D longitudinal beam profiles between the 2DBPGreer method and the 2DBPunlocked, 2DBP, and MapCHECKTR methods, respectively; a 5 mm positioning error is intentionally introduced in the calibration procedures of the latter three methods. CAX = central axis.

As shown in Table 1, when a 5 mm positioning error was introduced into the calibration procedures of the 2DBPunlocked, 2DBP proposed method, and the MapCHECKTR method, the absolute differences are 3.33%±1.82%,0.40%±0.36%, and 3.58%±1.94%, respectively. The 2D difference images and corresponding histograms of the above three methods are shown in Fig. 5, from top to bottom rows. Up to 7% differences are observed in the histograms of the 2DBPunlocked and MapCHECKTR method. The 1D CAX longitudinal beam profiles are plotted in Fig. 6. According to the proposed error model, the output variation and the positioning inaccuracy lead to 0.0% and 0.28% local error contribution, respectively, and these errors accumulate to around 5.0% propagation error at the beam edges.

**Figure 7:**
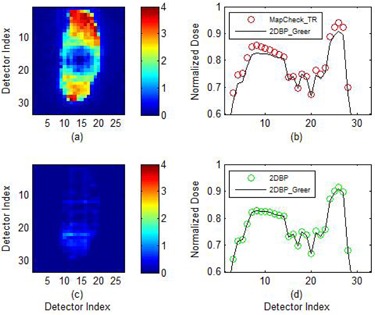
Comparisons of the sensitivity‐corrected IMRT fields between the 2DBPGreer method and MapCHECKTR and 2DBP method respectively; a 5mm positioning error is intentionally introduced in the calibration procedures of the latter two methods. (a) up to 3.9% 2D percentage differences between fields corrected by 2DBPGreer and MapCHECKTR method; (b) the central longitudinal profiles; (c) up to 1.4% 2D percentage differences between fields corrected by 2DBPGreer and 2DBP method; (d) the central longitudinal profiles.

A 5 mm positioning error was also introduced in the calibration procedures of the 2DBP and the MapCHECKTR method for the patient study. Comparisons were presented in Fig. 7 for the field “7 G160”. It suggests that the MapCHECKTR calibrated field deviates up to 3.9% from the 2DBPGreer field and the 2DBP method reduces that deviation to 1.4%. When the DTA analysis was conducted for all seven patient fields (Table 2), it was found that the MapCHECKTR method resulted in a higher DTA passing rate for the field “5 G80” than the 2DBP and 2DBPGreer method. For all other fields, the MapCHECKTR method resulted in the DTA passing rate −2.7714%±2.7269% lower than the 2DBPGreer method; the 2DBP method resulted in the DTA passing rate 0.3%±0.29% higher.

**Table 2:** DTA analysis of measured patient fields corrected by different calibration methods with the planar doses exported from Pinnacle treatment planning systems; a 3 mm DTA criteria was used. Moreover, a 5 mm positioning error is intentionally introduced in the calibration procedures of the 2DBP and MapCHECKTR methods.

*Field Name*	*2DBPGreer (%)*	*2DBP (%)*	*MapCHECKTR (%)*
1 G200	97.7	98.2	94.9
2 G320	98.0	98.5	97.3
3 G0	95.8	96.5	92.2
4 G40	94.4	94.8	93.2
5 G80	86.5	89.0	94.6
6 G120	98.4	98.4	95.5
7 G160	97.5	97.5	89.3

## IV. DISCUSSION

Once the thickness of the water slab is determined in the SW approach, the measurement is fairly easy; only one irradiation is required. This is also true for the 1DMCF approach after the beam profile is fitted in terms of MC‐simulations. However, those approaches require sophisticated and precise modeling of all components in the beam delivery systems and running time‐consuming MC simulations. On top of that, the parameters of MC simulations are generally tuned to match the in‐water measurements;^(8)^ when the investigated detector has a different composition from water, it is very likely the MC simulations have to be re‐run for that type of detector. The complexity of MC simulations makes the above approaches difficult to implement for inexperienced users. Also, the required a priori knowledge of the linac and detector array, as well as computation resources, might not come in handy to every user. Those methods also do not account for the machine variation between modeling phase and measurement phase.

The calibration quality of the MF method depends on the fluence uniformity of the field. As shown in the extracted beam profile of 6 MV photon beam by MapCHECK2 (Fig. 4), the maximum variation of the beam profile within $10\times 10\;{\text{cm}}^{2}$ (the field size originally used by this method) is around 3%. Even without considering other potential errors, MF method will inherently introduce up to 3% error in the calibrated sensitivities. The inherent error could be reduced if a smaller field size is used. However, this will increase the number of calibration fields.

In aforementioned three methods, the beam profile needs to be known ahead of time or to be modified. This is not required for 2DBP, 2DBPGreer, or MapCHECKTR method. Theoretically beam profile of any shape can be used in these methods. However, there are less variation and lower gradient in the open‐field beam profile. The usage of open field helps reduce the fluence variation due to positioning error. In recent years, linear accelerators with flattening filter‐free (FFF) beams have been introduced in many radiation oncology departments. As a flattening filter is not used, the profile of FFF beams is peaked at the center. The 2DBP, 2DBPGreer or MapCHECKTR methods can be used for the FFF beams without any modifications as they are independent of the shape of the beam, while it would be difficult, if not totally impossible, to adapt SW, 1DMCF, and MF methods. Due to the limitations of the latter three methods, this study has been focused on 2DBP, 2DBPGreer, and MapCHECKTR methods.

The output variation of linac affects the calibration accuracy of 2DBP, 2DBPGreer or MapCHECKTR methods. However, the output variation only leads to local calibration errors in 2DBPGreer method as the sensitivity at specific location from CAX is determined by a specific pair of narrow fields and the sensitivity is independent of those at other off‐axial locations. In contrast, since both 2DBP and TR methods employ iterative operations, the local error propagates and accumulates. The positioning error also leads to deviations from the expected fluence and, consequently, affects the calibration. Again, the impact of this type of error is more prominent for 2DBP and MapCHECKTR methods due to the iterative nature of these methods. A good example is presented in Fig. 4; the local output and positioning errors in the 2DBP implementation were estimated to be 0.036% and 0.054%, respectively. These errors would result in tiny perturbations in the 2DBPGreer method. However, these same tiny errors can end up with 1.45% and 0.3% deviations in the iterative method such as the 2DBP method.

There is a sophisticated mechanism in the MapCHECKTR method to remove the propagation error due to output variation with a 180° rotation measurement. In addition, the nonuniform perturbations due to linac beam symmetry variations and insufficient sidescatter were studied by Simon et al.^(9)^ His results suggested taking these situations into consideration would further reduce the calibration error from ±1.6% to ±0.48%. Yet, the positioning error is still not modeled in the MapCHECKTR method. As demonstrated in Fig. 6, a 5 mm positioning error leads to about 5% propagation error in the MapCHECKTR calibration results. When the error of similar magnitude is introduced into the 2DBP method, it can be substantially reduced by the proposed error‐locking mechanism. The patient study with this 5 mm positioning error also suggested the 2DBP method resulted in comparable DTA passing rates to the reference 2DBPGreer method, while the MapCHECKTR method resulted in an on‐average 2.7% lower DTA passing rate. In the patient study, the results of the field “5 G80” were not in agreement with the trends shown in other fields. After examining the field, it was found that the field is highly modulated with a high gradient fluence distribution. The odd behavior of the DTA analysis is due to the imperfect TPS (treatment planning system) modeling of the planar dose.

The positioning error of 5 mm is chosen in the experiments because the offset is in between two detectors and is easily discernible. More realistic positioning error under normal operation condition can be estimated from the reconstructed beam profile. In Fig. 6, it can be seen that the relative beam profile is 1.08 at 15 cm from CAX. The error introduced by 1 mm displacement is 0.08/150. The propagation error after 16 iterations (as in longitudinal beam profile reconstruction) is $(1+0.08/{150)}^{16}=1.0086$, that is, 0.86% deviation from the ideal case; similarly, 2 mm positioning error leads to a 1.72% deviation. Moreover, the positioning error might be introduced when an absent‐minded operator manually aligns the detector array (such as MapCHECK) or a robotically operated array (such as EPID) undergoes mechanic failure. In these cases, the calibration result could be egregiously wrong if the positioning error is not detected and removed.

As shown by Simon and colleagues,^(9)^ the output variation from 10 repeated irradiations of the Elekta linac was less than 0.2% and it is even less for Varian linac. With careful alignment of the detector array and normal performance of linac systems, one should not see substantial propagation error in the calibration results. That said, an error‐locking scheme should still be incorporated into an iterative method since small unexpected errors might be amplified to an unacceptable level via iteration operations, and the robustness of a calibration method should not completely rely on the stability of linac systems and the reliability of the operator. In addition, the error‐locking scheme also provides a clue of potential calibration error. For instance, in Fig. 6, the calculated 0.28% positioning error and 0.0% output error clearly indicated that a major positioning error had occurred during the calibration procedure.

The energy dependence of the sensitivity isn't considered in this study. For instance, the energy spectra used to calculate the sensitivity of an off‐axial detector in the MapCHECKTR method is from the off‐axial location of a wide beam, while it is from the CAX of a narrow field in the 2DBPGreer and 2DBP methods. The results of this study did show a 0.84%±0.60% difference between the MapCHECKTR and 2DBPGreer methods. This difference can be attributed to the positioning error (not handled by the MapCHECKTR method) or the energy spectra variation. It is out of scope of this study to differentiate them, but the latter contribution might be small since modern diode is made with less energy dependence.

As rotation operation is not needed for the proposed method, it is applicable to the calibration of in‐line detector array such as EPID. The method was not validated on the EPID as the robotic arm of the imager introduces backscattered radiation,^(10)^ which changes fluence of a wide beam irradiation at the unshifted and shifted positions of the proposed procedure. This significant variation of fluence is not acceptable for the iterative method due to error propagation. Progress has been made to alter the hardware of the imager to eliminate the backscattered radiation.^(11)^ Therefore, one can expect to apply the proposed method on EPID in the near future.

## V. CONCLUSIONS

Among all reviewed array calibration methods in this study, the measurement‐based iterative method is of greatest interest as it does not require complex modeling and the number of measurements is minimal. One of such sensitivity calibration methods has been proposed in this study and validated on the MapCHECK2 devices. An error‐locking scheme has been proposed to reduce the propagation errors due to intermeasurement output variation and positioning inaccuracy. The calibration results of the proposed method are in close agreement with those by the reference noniterative method and by the commercial method. Compared to the commercially available iterative method, the proposed method does not need rotation of the detector array and can be used for in‐line array such as EPID. More importantly, the error‐locking scheme is able to remove the calibration error from inaccurate positioning that can't be handled by the commercial method and, therefore, significantly increases the robustness of the proposed method.

## Supporting information

Supplementary Material

Supplementary Material
